# Gut microbiome perturbation, antibiotic resistance, and *Escherichia coli* strain dynamics associated with international travel: a metagenomic analysis

**DOI:** 10.1016/S2666-5247(23)00147-7

**Published:** 2023-09-13

**Authors:** Colin J Worby, Sushmita Sridhar, Sarah E Turbett, Margaret V Becker, Lucyna Kogut, Vanessa Sanchez, Ryan A Bronson, Sowmya R Rao, Elizabeth Oliver, Allison Taylor Walker, Maroya Spalding Walters, Paul Kelly, Daniel T Leung, Mark C Knouse, Stefan H F Hagmann, Jason B Harris, Edward T Ryan, Ashlee M Earl, Regina C LaRocque

**Affiliations:** Infectious Disease and Microbiome Program, The Broad Institute of MIT and Harvard, Cambridge, MA, USA (C J Worby PhD, R A Bronson MS, A M Earl PhD); Department of Medicine, Harvard Medical School, Boston, MA, USA (S Sridhar PhD, Prof S E Turbett MD, Prof E T Ryan MD, Prof R C LaRocque MD MPH); Division of Infectious Diseases (S Sridhar, Prof S E Turbett, M V Becker BS, L Kogut BS, V Sanchez BS, E Oliver RN, Prof J B Harris MD, Prof E T Ryan, Prof R C LaRocque), Department of Pathology (Prof S E Turbett), and Travellers’ Advice and Immunization Center (Prof E T Ryan, Prof R C LaRocque), Massachusetts General Hospital, Boston, MA, USA; Department of Global Health, Boston University School of Public Health, Boston, MA, USA (S R Rao PhD); Division of Global Migration and Quarantine (A T Walker PhD) and Division of Healthcare Quality Promotion, National Center for Emerging and Zoonotic Infectious Disease (M S Walters PhD), Centers for Disease Control and Prevention, Atlanta, GA, USA; Division of Infectious Diseases, Bronx Care Center, Bronx, NY, USA (P Kelly MD); Division of Infectious Diseases and Division of Microbiology and Immunology, University of Utah, Salt Lake City, UT, USA (D T Leung MD); Department of Medicine, Lehigh Valley Health Network, Allentown, PA, USA (M C Knouse MD); Division of Pediatric Infectious Diseases, Steven and Alexandra Cohen Children’s Medical Center of New York/Northwell Health, New Hyde Park, NY, USA (S H F Hagmann MD); Division of Pediatric Global Health, Massachusetts General Hospital for Children, Boston, MA, USA (Prof J B Harris)

## Abstract

**Background:**

Culture-based studies have shown that acquisition of extended-spectrum β-lactamase-producing Enterobacterales is common during international travel; however, little is known about the role of the gut microbiome before and during travel, nor about acquisition of other antimicrobial-resistant organisms. We aimed to identify (1) whether the gut microbiome provided colonisation resistance against antimicrobial-resistant organism acquisition, (2) the effect of travel and travel behaviours on the gut microbiome, and (3) the scale and global heterogeneity of antimicrobial-resistant organism acquisition.

**Methods:**

In this metagenomic analysis, participants were recruited at three US travel clinics (Boston, MA; New York, NY; and Salt Lake City, UT) before international travel. Participants had to travel internationally between Dec 8, 2017, and April 30, 2019, and have DNA extractions for stool samples both before and after travel for inclusion. Participants were excluded if they had at least one low coverage sample (<1 million read pairs). Stool samples were collected at home before and after travel, sent to a clinical microbiology laboratory to be screened for three target antimicrobial-resistant organisms (extended-spectrum β-lactamase-producing Enterobacterales, carbapenem-resistant Enterobacterales, and *mcr*-mediated colistin-resistant Enterobacterales), and underwent DNA extraction and shotgun metagenomic sequencing. We profiled metagenomes for taxonomic composition, antibiotic-resistant gene content, and characterised the *Escherichia coli* population at the strain level. We analysed pre-travel samples to identify the gut microbiome risk factors associated with acquisition of the three targeted antimicrobial resistant organisms. Pre-travel and post-travel samples were compared to identify microbiome and resistome perturbation and *E coli* strain acquisition associated with travel.

**Findings:**

A total of 368 individuals travelled between the required dates, and 296 had DNA extractions available for both before and after travel. 29 travellers were excluded as they had at least one low coverage sample, leaving a final group of 267 participants. We observed a perturbation of the gut microbiota, characterised by a significant depletion of microbial diversity and enrichment of the *Enterobacteriaceae* family. Metagenomic strain tracking confirmed that 67% of travellers acquired new strains of *E coli* during travel that were phylogenetically distinct from their pre-travel strains. We observed widespread enrichment of antibiotic-resistant genes in the gut, with a median 15% (95% CI 10–20, p<1 × 10^−10^) increase in burden (reads per kilobase per million reads). This increase included antibiotic-resistant genes previously classified as threats to public health, which were 56% (95% CI 36–91, p=2 × 10^−^11) higher in abundance after travel than before. Fluoroquinolone antibiotic-resistant genes were aquired by 97 (54%) of 181 travellers with no detected pre-travel carriage. Although we found that visiting friends or relatives, travel to south Asia, and eating uncooked vegetables were risk factors for acquisition of the three targeted antimicrobial resistant organisms, we did not observe an association between the pre-travel microbiome structure and travel-related antimicrobial-resistant organism acquisition.

**Interpretation:**

This work highlights a scale of *E coli* and antimicrobial-resistant organism acquisition by US travellers not apparent from previous culture-based studies, and suggests that strategies to control antimicrobial-resistant organisms addressing international traveller behaviour, rather than modulating the gut microbiome, could be worthwhile.

## Introduction

Antimicrobial resistance (AMR) is an urgent threat to global public health.^1^ Antimicrobial-resistant (AMR) organisms are associated with over 2.8 million infections and 35 000 deaths per year in the USA alone.^2^ Multiple factors are associated with increasing prevalence of AMR, and the ease with which resistant organisms and AMR-associated genes can disperse makes antimicrobial resistance a global problem.^3^ Novel AMR organisms can disseminate rapidly around the world,^4^ limiting the effectiveness of local efforts to mitigate the prevalence of resistance.

International travel is a known facilitator of the global spread of AMR.^5^ Extended-spectrum β-lactamase (ESBL)-producing organisms are considered a serious threat,^2^ and are acquired by approximately 30% of studied travellers;^6^ antibiotic usage, travellers’ diarrhoea, and travel destination are recognised risk factors.^5^ Acquired ESBL-producing organisms can persist in the gut after return from travelling,^7^ posing a risk for subsequent infection^8^ and ongoing transmission.^9^

Acquisition of AMR organisms requires exposure to relevant microbial reservoirs and successful colonisation by the organism, with geographical and host factors both having a role. Geographical factors probably reflect local AMR prevalence, whereas travellers’ diarrhoea and antibiotic use are factors that could increase susceptibility to colonisation. The gut microbiota are increasingly recognised as a source of colonisation resistance; perturbation of a healthy gut microbiome can diminish this effect.^10^ Because diarrhoea^11^ and antibiotic use^12^ affect gut microbes, their association with acquisition of ESBL-producing organisms might relate to diminished colonisation resistance. Previous studies suggest that the pre-travel microbiome could modulate susceptibility to travellers’ diarrhoea^13^ or ESBL-producing organism colonisation,^14^ but these studies were small and based on amplicon sequencing, rather than whole-metagenome sequencing. Previous culture-based studies have identified travel-associated acquisition of carbapenem-resistant and *mcr*-mediated colistin-resistant organisms in addition to ESBL-producing organisms,^15^ while whole-metagenomic shotgun sequencing has been used to show widespread AMR acquisition during travel;^16^ however, the organisms associated with this AMR burden have yet to be well characterised.

Here, we use stool samples collected from a large cohort of US international travellers to evaluate the role of the pre-travel gut microbiome in susceptibility to AMR organism acquisition and the effect of travel on the gut microbiota, the total burden of AMR-associated genes (referred to as the resistome), and *Escherichia coli* strain carriage.

## Methods

### Study design and participants

In this metagenomic analysis, we recruited US international travellers at three travel clinics (Boston, MA; New York, NY; and Salt Lake City, UT) affiliated with Global TravEpiNet, a pre-travel health-care consortium,^17^ as part of a previously described study.^15^ Study approval was obtained from institutional review boards at each participating site. Recruitment and sample processing began on Nov 27, 2017, at the Boston site; on Aug 15, 2018, at the New York site; and on Sept 10, 2018, at the Salt Lake City site, and were done on a rolling basis. We implemented a cutoff date of April 30, 2019, for inclusion in this metagenomic analysis at all three sites.

Participants were required to have travelled internationally between the study recruitment dates and to have DNA extractions available for stool samples from both before and after travel. Individuals who had at least one low coverage sample (<1 million read pairs) were excluded. Research staff approached travel clinic visitors to offer participation in the study. Health-care providers used a structured questionnaire to collect demographic information, clinical histories, and travel details; no inclusion or exclusion criteria were specified including for age. Participants or their guardians provided written informed consent. Institutional review board approval was obtained at each of the participating sites.

### Procedures

Travellers collected stool samples before departure and after return and completed a post-travel questionnaire regarding travel-related behaviours and illnesses (appendix 1 p 4). Details on ethnicity were not collected. Participants self-collected a stool sample, putting separate portions into ethanol and into Cary-Blair medium (Thermo Fisher Scientific; Lenexa, KS, USA). Samples were posted to the laboratory at Massachusetts General Hospital in accordance with biosafety standards.^18^ Samples transported in Cary-Blair medium were screened for the presence of three target AMR organisms: ESBL-producing Enter obacterales, carbapenem-resistant Enterobacterales, and *mcr*-mediated colistin-resistant Enterobacterales, using US Food and Drug Administration-approved or internally validated culture-based methods, as previously described.^18,19^ Ethanol samples were stored at −80°C until DNA extraction and metagenomic sequencing.

DNA extraction was done with the PowerFecal DNA/RNA kit (QIAGEN; Hilden, Germany). We used a NovaSeq 6000 system (Illumina; San Diego, CA, USA) with 151 bp paired-end reads to yield a median of 14 million paired-end reads per sample for sequencing. Metagenomic libraries were prepared using the Nextera XT DNA Library Preparation kit (Illumina) and data were processed with the Broad Picard Pipeline. Further details on DNA extraction, sequencing, and metagenomic analysis are described in appendix 1 (p 2). *E coli* strain content was characterised using StrainGE (version 1.1.4).^20^ Resistance gene content was profiled using ShortBRED (version 0.9.5),^21^ with AMR-associated genes classified as high risk if they were classified as “Rank I - current threat” by Zhang and colleagues.^22^ Using the vegan (version 2.6–4) package in R, microbiome diversity was quantified with the Shannon index, and perturbation was measured as the Bray Curtis index between the pre-travel and post-travel samples, using the vegdist function.

### Statistical analysis

The sampled cohort represents a convenience sample; as such, no power calculations were done. Differences in continuous measures (ie, microbial diversity, relative abundance, and Bray Curtis dissimilarity) before and after travel were tested using a paired *t* test or Wilcoxon signed rank test. Differences in count measures (ie, number of *E coli* strains) before and after travel were compared using an exact Poisson test. Exact binomial tests were used to assess the non-randomness of changes (eg, increase *vs* decrease in abundance). Traveller questionnaire responses were categorised into travel factors (eg, destination, duration, and enrolment site), host factors (eg, medications, multivitamins, probiotics, medical conditions, pre-travel vaccinations, antibiotic exposure, or hospital admission in the preceding year, and diarrhoea during travel), and behavioural factors (eg, travel purpose and consumption of undercooked or unwashed food or unfiltered water; appendix 2). We fit regression models to identify the effect of these factors on the following outcomes in all participants: targeted AMR organism acquisition, travellers’ diarrhoea, gut diversity, gut microbiome perturbation, taxa relative abundances, *E coli* strain acquisition, and AMR-associated gene burden (appendix 1 p 2).

We considered acquisition of targeted AMR organisms together rather than ESBL-producing Enterobacterales, carbapenem-resistant Enterobacterales, and *mcr*-mediated colistin-resistant Enterobacterales individually, since acquisition of carbapenem-resistant Enterobacterales, and *mcr*-mediated colistin-resistant Enterobacterales was rare, and only two individuals acquired a targeted AMR organism without also acquiring an ESBL-producing Enterobacterales. We report odds ratios (ORs) and 95% CIs for all outcomes, with the exception of microbiome perturbation, where we estimate the change in Bray Curtis dissimilarity. Benjamini-Hochberg correction was applied to adjust for multiple hypothesis testing, with a two-sided threshold for significance of p<0·05.

### Role of the funding source

The study funders collaborated with co-investigators to design the study and were involved in data interpretation and writing of the manuscript. The study funders had no involvement in data collection or data analysis.

## Results

Of the 608 travellers comprising the previously described full cohort,^15^ 368 had completed travel by the cutoff date, and DNA extractions were available for both pre-travel and post-travel samples for 296 travellers. 29 travellers were excluded as they had at least one low coverage sample. We studied the pre-travel and post-travel faecal metagenomes of 267 travellers (table).^15^ Post-travel stool samples were collected a median of 11 days (IQR 7–16) after return. A total of 101 (38%) travellers acquired at least one of the three targeted AMR organisms, based on culture; 99 (98%) of these travellers acquired an ESBL-producing organism, 18 (18%) acquired *mcr*-mediated colistin-resistant Enterobacterales, and 3 (3%) acquired carbapenem-resistant Enterobacterales. All travellers acquiring an ESBL-producing organism acquired an ESBL-producing *E coli*; three (3%) additionally acquired ESBL-producing *Klebsiella pneumoniae*. 88 (33%) of 267 travellers reported travellers’ diarrhoea, of whom 16 (18%) reported antibiotic treatment (ciprofloxacin n=7 or azithromycin n=9). An additional 14 (5%) travellers took antibiotics (including doxycycline n=6) for other reasons.

To determine gut microbiome risk factors for acquisition of the targeted AMR organisms, we first fit logistic regression models with acquisition as a function of travel, host, and behavioural risk factors (appendix 2 p 1). Adjusting for selected dietary factors, we found visiting friends or relatives (OR 4·15, 95% CI 1·23–16·79, p=0·029), travel to south Asia (2·34, 1·06–5·32, p=0·037), and eating uncooked vegetables (2·19, 1·21–4·04, p=0·011) to be risk factors for acquisition of the targeted AMR organisms, whereas travel to southern Africa was associated with reduced acquisition (0·15, 0·02–0·55, p=0·014; appendix 2 p 2). Adjusting for these factors, we found that pre-travel gut diversity had no effect on the risk of acquisition of the targeted AMR organisms (0·96, 0·78–1·17, p=0·68). In addition, we did not find the pre-travel relative abundance of any taxon to be associated with differential risk of acquisition for the targeted AMR organisms. We repeated this analysis with travellers’ diarrhoea as an outcome; eating street food (3·10, 1·51–6·55, p=0·0024) and antibiotic use (3·38, 1·46–7·95, p=0·0045) were risk factors (appendix 2 p 3). No pre-travel microbiome factors were associated with risk of travellers’ diarrhoea during travel.

**Figure 1:**
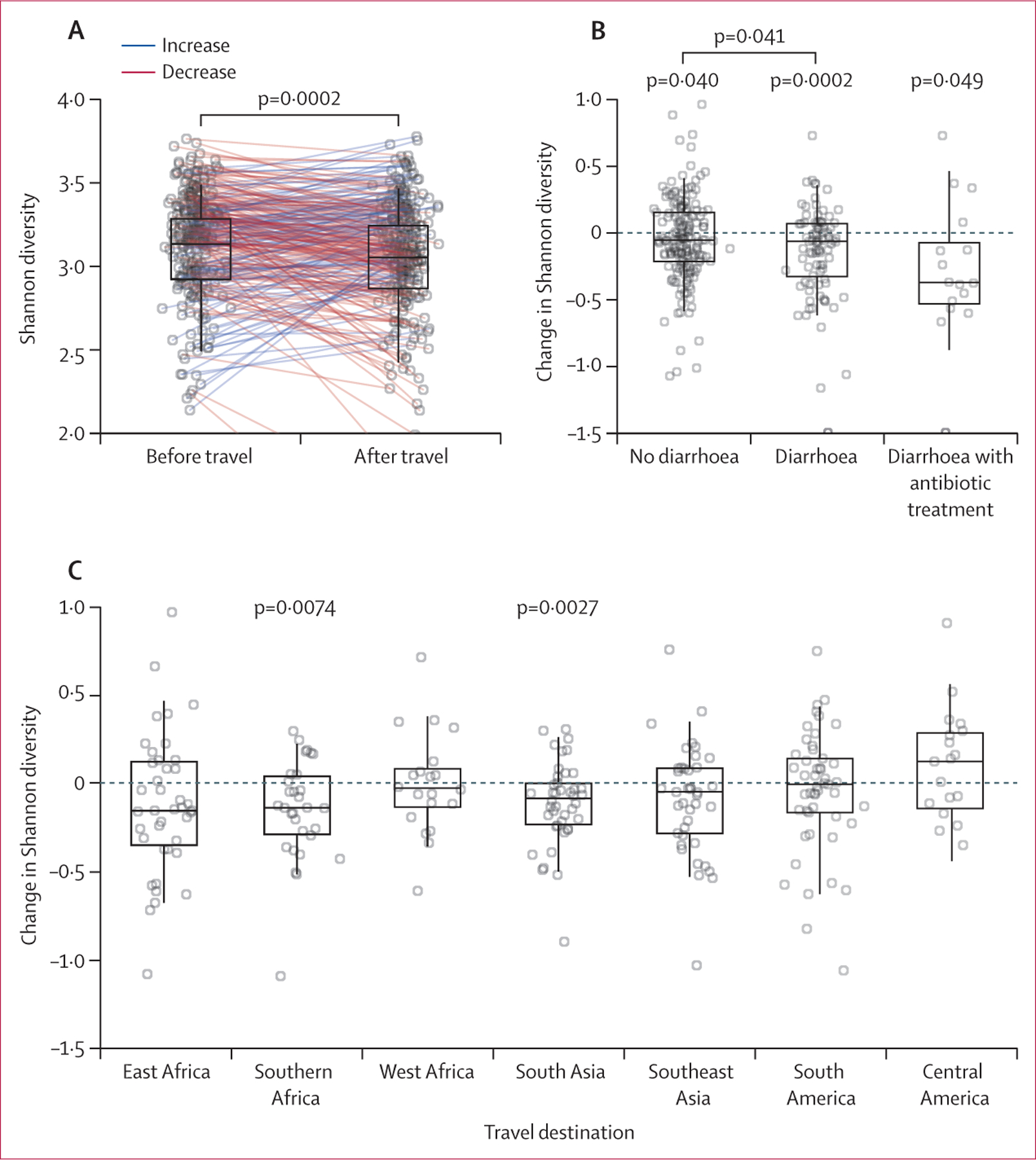
Travel-associated loss of diversity (A) Shannon diversity of pre-travel and post-travel samples, with sample pairs from the same traveller linked by a line. (B) The absolute change in Shannon diversity observed in travellers with and without travellers’ diarrhoea, and those reporting antibiotic treatment for travellers’ diarrhoea. (C) Absolute change in Shannon diversity associated with travel to the eight most common travel destinations in the study. All box plots denote the median, IQR, and 95% quantiles. Significance was tested by *t* tests (paired except for between-group comparisons).

164 (61%) of 267 travellers had a loss of microbial diversity after travel (change in Shannon diversity −0·076, 95% CI −0·036 to −0·115, paired *t* test p=0·0003; figure 1A). The proportion of travellers with a loss of gut microbial diversity differed by region of travel, ranging from 42% (8/19) among travellers to central America to 76% (32/42) among travellers to south Asia (figure 1C). Travellers reporting travellers’ diarrhoea had a greater reduction in microbial diversity upon return compared with travellers without travellers’ diarrhoea (difference in reduction −0·09, −0·05 to −0·14, *t* test p=0·041); travellers who took antibiotics to treat travellers’ diarrhoea had the greatest loss of diversity (figure 1B). Loss of gut microbial diversity did not differ between travellers who acquired a targeted AMR organism and those who did not (difference in reduction −0·01, −0·09 to 0·07, p=0·81 *t* test).

Travel was also associated with shifts in taxonomic relative abundance, or gut microbiome perturbation, which did not affect overall diversity. Antibiotic use was associated with a significant perturbation of the gut microbiome (change in Bray Curtis dissimilarity 0·06, 95% CI 0·02 to 0·11, p=0·0046), whereas consumption of unfiltered tap water was unexpectedly associated with significantly smaller perturbations, meaning greater microbiome stability (−0·04, −0·07 to −0·01, p=0·015). These results remained significant after adjusting for selected dietary and travel behaviours (appendix 2 p 4). The perturbation among travellers who acquired a targeted AMR organism was no different than among those who did not (0·02, −0·01 to 0·05, p=0·16).

**Figure 2:**
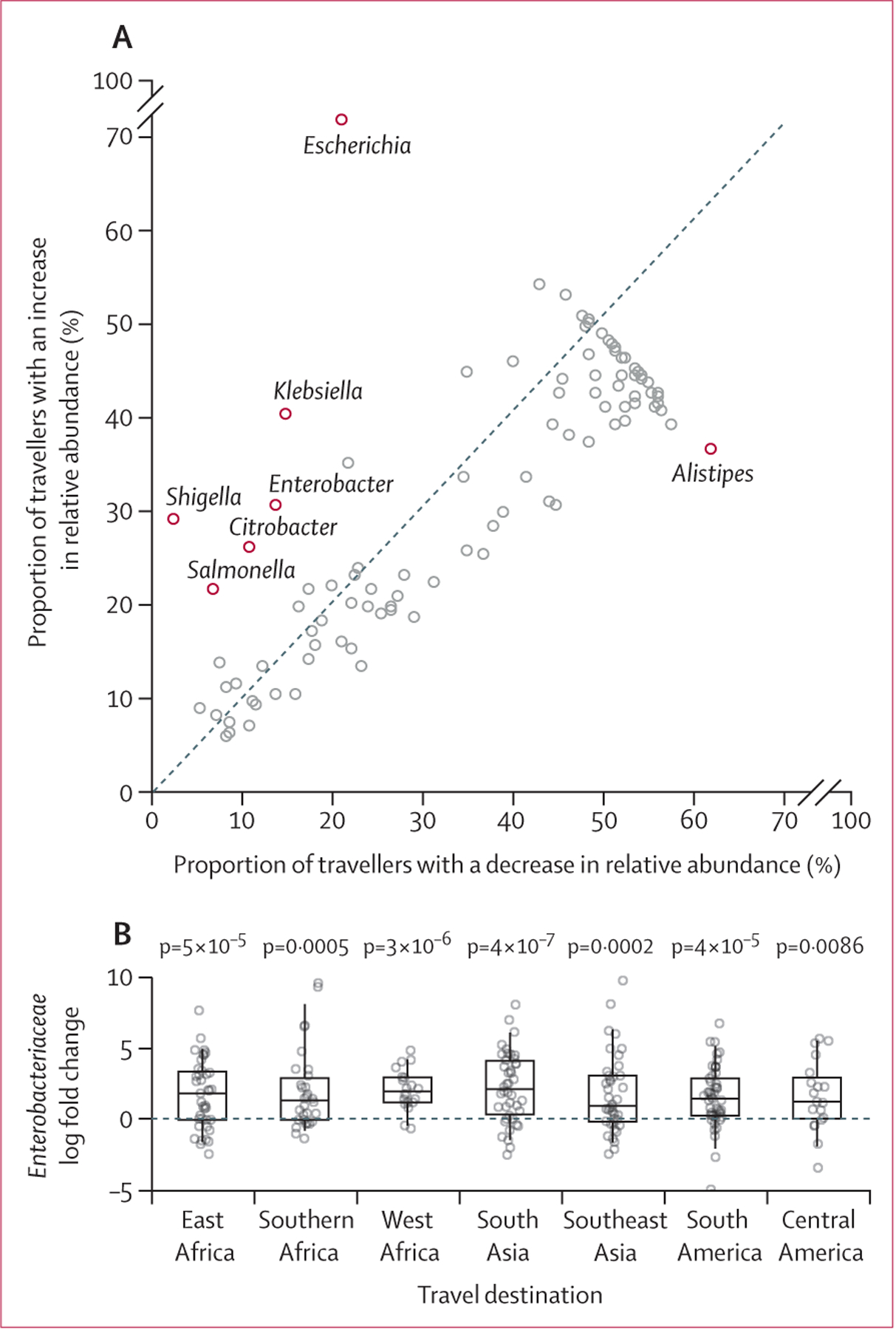
Surge in *Enterobacteriaceae* associated with international travel (A) For each genus present in at least 10% of samples, the proportion of travellers with an observed decrease *vs* increase in relative abundance. Red labelled points denote genera with significant skew. (B) The travel-associated log fold change in *Enterobacteriaceae* observed in travellers visiting each of the eight most common destination regions. All box plots denote the median, IQR, and 95% quantiles. Significance determined by paired *t* tests.

The abundance of *Escherichia* spp was elevated in a significant proportion of travellers after travel relative to before travel (figure 2A), with overall median relative abundance increasing from 0·1% (IQR 0·0–0·35) to 0·6% (0·1–3·0; Wilcoxon signed rank test p<1 × 10^−10^). Several related genera within the *Enterobacteriaceae* family were also significantly elevated after travel, including *Klebsiella*, *Enterobacter*, and *Salmonella* (figure 2A). Conversely, the genus *Alistipes* (*Rickenellaceae* family) was observed to be depleted after travel. Similar patterns were observed in genus acquisition (defined as present in post-travel microbiomes but not pre-travel microbiomes); 33% of travellers acquired *Klebsiella* spp (*vs* 8% loss, p<1 × 10^−10^), 26% acquired *Shigella* spp (*vs* 1% loss, p<1 × 10^−10^), 25% acquired *Enterobacter* spp (*vs* 10% loss, p=0·0004), 24% acquired *Citrobacter* spp (*vs* 7% loss, p=1 × 10^−5^), and 22% acquired *Escherichia* spp (*vs* 5% loss, p=3 × 10^−6^; appendix 1 p 9).

This increase in *Enterobacteriaceae* was observed across most travel regions (figure 2B). We fit models to identify factors contributing to the change in relative abundance for each genus in *Enterobacteriaceae*. Travellers visiting friends or relatives had a greater increase in *Klebsiella* spp, *Enterobacter* spp, and *Citrobacter* spp abundance compared with those with other travel purposes (appendix 1 p 8). Beyond the Enterobacteriaceae family, we found that antibiotic use was associated with a significant depletion of several genera including *Faecalibacterium* spp, *Bifidobacterium* spp, and *Ruminococcus* spp, as well as an increase in *Lachnoclostridium* spp and *Flavonifractor* spp (appendix 1 p 8). Travellers’ diarrhoea was associated with a depletion of *Ruthenibacterium* spp.

**Figure 3:**
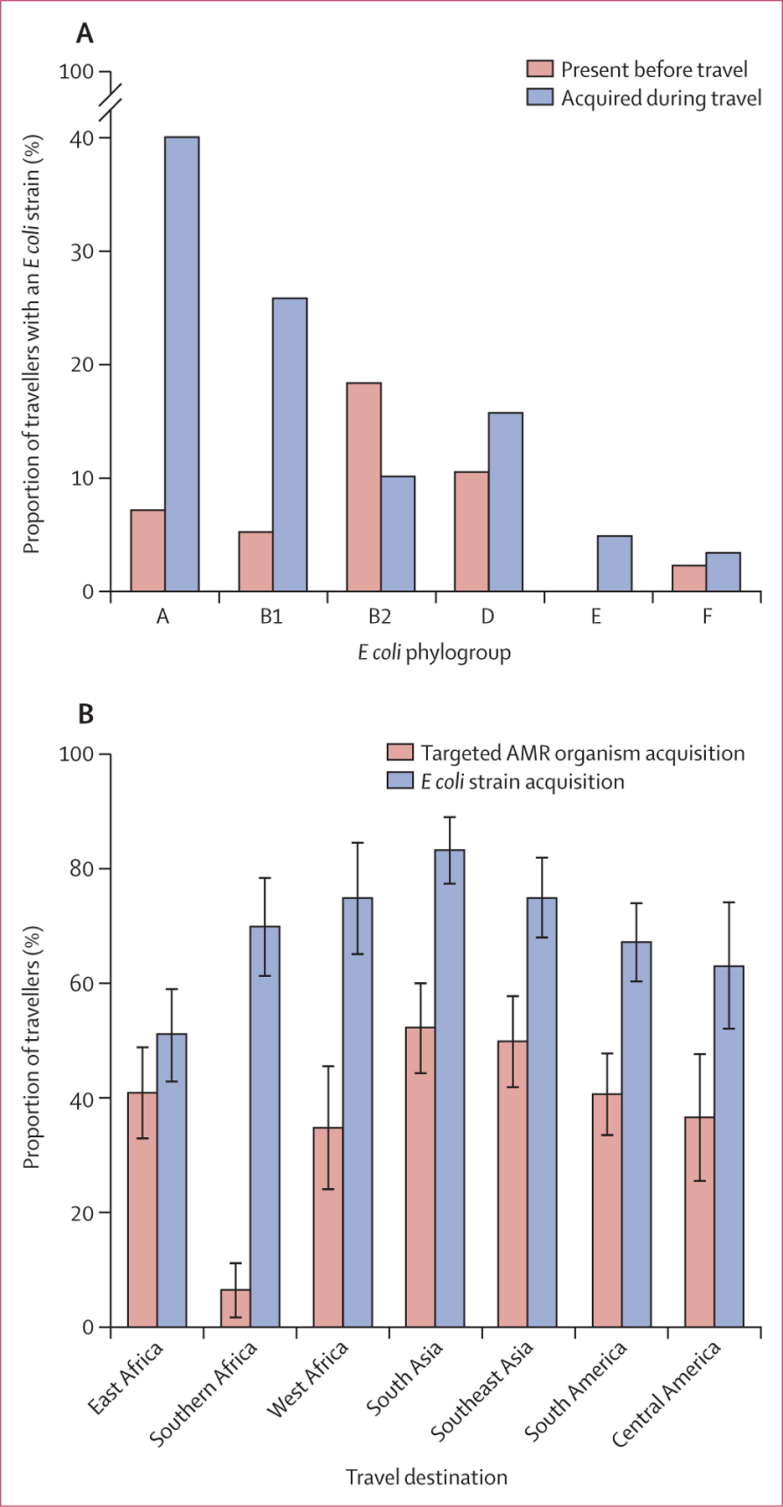
Travel-acquired *Escherichia coli* strains are phylogenetically distinct from those present pre-travel (A) For each *E coli* phylogroup with more than five observations, the proportion of travellers carrying a strain before travel is compared with the proportion of travellers acquiring a strain during travel. Acquisition is defined here as a strain detected in the post-travel sample that was not detected in the pre-travel sample. (B) The proportion of travellers visiting each travel destination who acquired a targeted AMR organism (based on culture; red) or at least one *E coli* strain (based on metagenomic analyses; blue). Error bars represent the standard errors of the proportions. AMR=antimicrobial resistant.

As the most frequently enriched genus, and the most common travel-acquired ESBL-producing organism,^15^ we sought to explore *Escherichia* dynamics at strain level, in particular to establish whether the elevated relative abundance represented acquisition of novel strains, or relative expansion of the pre-travel *Escherichia* population.^20^ We identified 447 *E coli* strains across the 534 samples; post-travel samples contained more strains (mean 1·2 strains per sample, SD 1·1) than did pre-travel samples (0·5, 0·72; exact Poisson test p<1 × 10^−10^), and most travellers returned with more strains than were present before travel (55% [146/267] *vs* 11% [29/267] returning with fewer strains; exact binomial test p<1 × 10^−10^). 178 (67%) of the 267 travellers acquired at least one new *E coli* strain, defined as a strain detected in the post-travel sample but not in the pre-travel sample. Of 187 travellers with an increase in *E coli* relative abundance, 159 (85%) acquired at least one *E coli* strain, compared with 19 (24%) of the 80 individuals who had no increase, suggesting that strain acquisition, rather than expansion of the pre-travel population, was driving the increase in *E coli* abundance after travel. Notably, acquired strains were phylogenetically distinct from those carried pre-travel; although most (83 [65%] of 127) pre-travel strains belonged to *E coli* phylogroups B2 and D, most acquired strains (215 [70%] of 306) belonged to phylogroups A and B1 (figure 3A). Phylogroup E, which was not detected in any pre-travel samples, was detected in 13 (5%) of 267 post-travel samples. The phylogroup distribution of acquired strains was broadly similar across travel destinations (appendix 1 p 10).

We fit logistic regression models for *E coli* strain acquisition with travel, host, and behavioural variables. Travel to south Asia (OR 2·88, 95% CI 1·29–7·34, p=0·016) was identified as a risk factor for acquisition, whereas consumption of unfiltered tap water was found to decrease the risk of *E coli* strain acquisition (0·47, 0·27–0·82, p=0·0083), as was pre-travel administration of any typhoid vaccine (0·44, 0·21–0·88, p=0·027). These associations remained significant in a multivariable model adjusting for additional selected dietary and travel factors (appendix 2 p 5). We partitioned typhoid vaccination into type; the identified negative association held for the intramuscular vaccine (0·34, 0·16–0·71, p=0·0051), but not the oral vaccine (0·49, 0·21–1·07, p=0·088). Although we found travel to south Asia to be a risk factor for both *E coli* and targeted AMR organism acquisition, risk factors were generally distinct for each outcome (appendix 2 pp 2, 5). 21 (70%) of the 30 travellers visiting southern Africa acquired *E coli* strains, yet only two (7%) acquired targeted AMR organisms (difference in proportions 0·63, 95% CI 0·41–0·85, χ2 test p=1·8 × 10^−6^; figure 3B).

**Figure 4:**
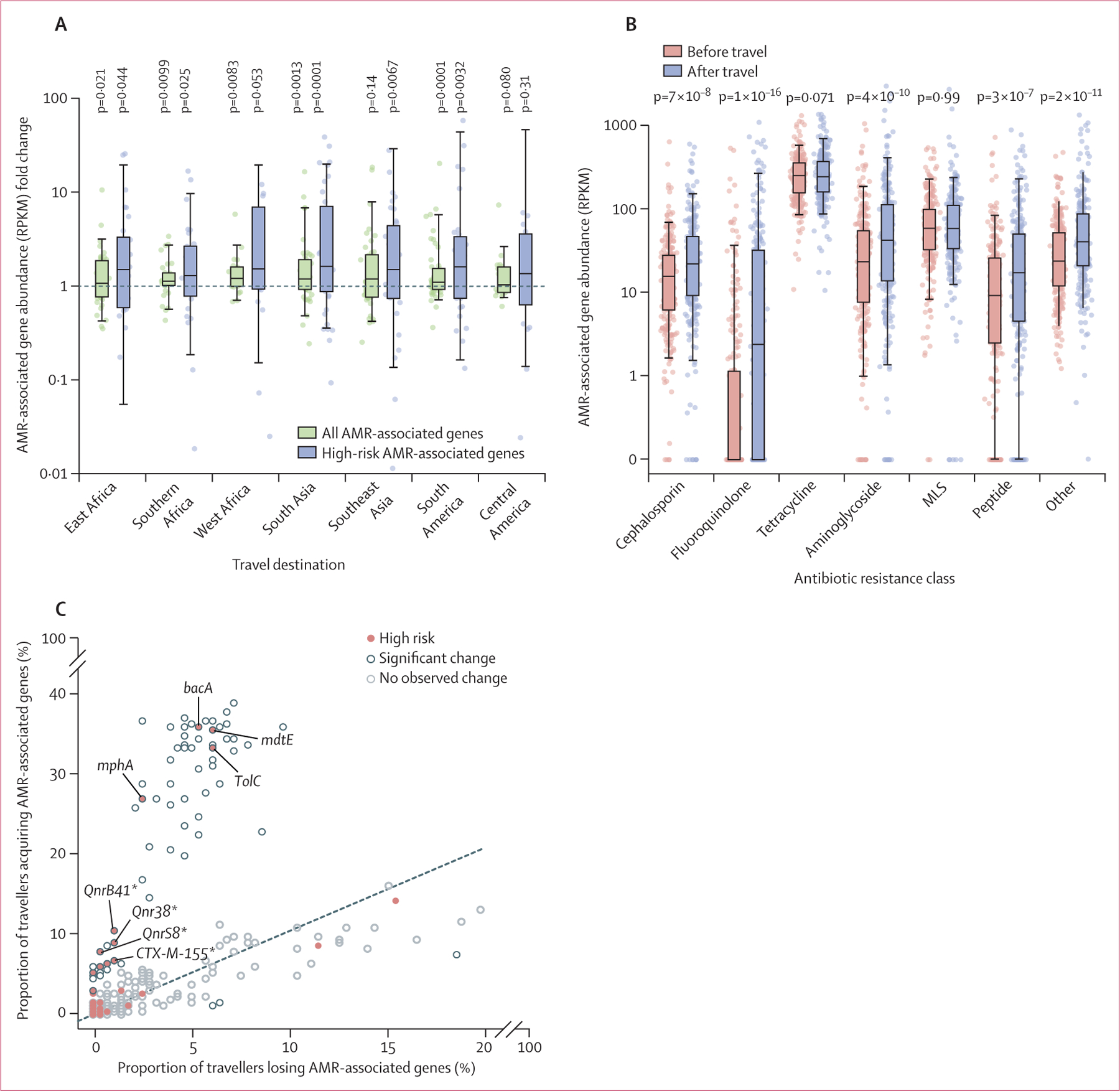
Acquisition of diverse AMR-associated gene during travel Box plots denote the median, IQR, and central 95% quantile. (A) Change in total and high-risk antibiotic-resistant gene abundance associated with travel. Significance was evaluated by Wilcoxon signed rank tests. (B) For seven common antibiotic-resistance classes, the pre-travel and post-travel abundance of AMR-associated genes are shown in RPKM. (C) For all AMR-associated genes detected, the proportions of travellers losing *vs* acquiring the AMR-associated genes are plotted against each other. Labelled AMR-associated genes are both significant and high risk (appendix 2 p 7). AMR=antimicrobial resistance. MLS=macrolides, lincosamides, streptogramines. RPKM=reads per kilobase per million reads. *Representatives of multi-gene groups based on identity clustering.

Metagenomic analysis allowed us to do an untargeted comparison of AMR before and after travel. Travel was associated with a median 15% (95% CI 10–20) increase in overall AMR-associated gene burden, measured as reads per kilobase per million reads (p<1 × 10^−10^ Wilcoxon signed rank test). Although travel to most destinations was associated with an increase in the overall AMR-associated gene burden, the magnitude of change varied by destination (figure 4A; appendix 2 p 6). The largest increases were observed among travellers visiting western Africa (median increase 27%, 95% CI 7–50) and south Asia (24%, 4–48).

The increase in AMR-associated gene burden was driven by several antibiotic classes (figure 4B). The gain in fluoroquinolone resistance was particularly notable; of the 181 travellers with no detected fluoroquinolone resistance determinants before travelling, 97 (54%) returned with at least one gene associated with resistance to an antibiotic in this class (exact binomial test p<1 × 10^−10^, with the null hypothesis being equal rate of antibiotic resistant gene loss). A total of 72 AMR-associated genes were acquired at significant rates (figure 4C; appendix p 7). Of these, 15 AMR-associated genes belonged to the highest public health risk category, based on previous classification,^22^ including genes encoding trimethoprim (*dfrA*) and fluoroquinolone (*qnrB* and *qnrS)* resistance and the ESBL-encoding gene cluster CTX-M group 1. Although high-risk AMR-associated genes were significantly enriched after travel among all travellers (median increase 56%, 95% CI 36–91, Wilcoxon signed rank test p=2 × 10^−11^), we observed the largest gains among those travelling to south Asia (92%, 51–158, p=0·0001), and south America (122%, 63–175, p=0·0032). Among travellers who did not acquire a targeted AMR organism, the increase in the burden of cephalosporin, fluoroquinolone, aminoglycoside, and peptide resistance remained significant, as did the increase in high-risk AMR-associated genes (40%, 10–68, p=0·0061). Although we could not definitively link specific AMR-associated genes to their bacterial host, 36 of the 50 most commonly acquired AMR-associated genes were strongly correlated with *E coli* relative abundance (appendix 2 p 7).

Finally, we compared our resistome data with results from a 2014 cohort of Dutch individuals who travelled to similar regions of the world as our study (appendix 1 p 11).^16,23^ Although largely concordant, we found that three (4%) of the 72 AMR-associated genes that were significantly elevated in our study did not increase significantly in abundance in the earlier Dutch study: two high-risk fluoroquinolone resistance-associated genes, *qnrB59* and *qnrB41*, and the polymyxin resistance gene *pmrE*. Conversely, seven (9%) of the 76 significantly elevated AMR-associated genes in the Dutch study did not increase significantly in abundance in our study: two high-risk β-lactamase-encoding genes, *OXA-31* and *CTX-M-110*, and *cepA*, *LAP-2*, *lsaE*, *AAC(3)-IIa*, and *SAT-2*.

## Discussion

We found that international travel, across a range of destinations and a variety of travel, host, and behavioural factors, is associated with a perturbation of the gut microbiome characterised by a reduction in microbial diversity, a surge in the abundance of *Enterobacteriaceae* organisms, and acquisition of multiple AMR-associated genes, including several that have previously been identified to pose the greatest risk to public health.^22^ Although a third of travellers acquired ESBL-producing organisms during travel, over two-thirds acquired new *E coli* strains. Previous culture-based studies have shown that travel to some regions, including south and southeast Asia, are associated with an elevated risk of acquiring ESBL-producing organisms.^5^ Our untargeted metagenomic approach shows that ESBL-producing organisms represent only a fraction of travel-acquired AMR organisms, and that similar geographical heterogeneity exists in the risk of acquiring other AMR-associated genes that pose a public health risk.

Notably, we did not find any microbiome risk factors to predict targeted AMR organism acquisition or travellers’ diarrhoea based on pre-travel samples, which contrasts with the results of two previous, smaller studies.^13,14^ Our larger study allowed us to account for a wide range of potential confounders, and we conclude that the pre-travel microbiome probably plays a considerably lesser role than the travel, host, and behavioural risk factors identified here. However, it is possible that perturbations to the microbiome during travel facilitate targeted AMR organism acquisition. We note that antibiotic use and diarrhoea, two frequently identified risk factors for acquisition of the targeted AMR organisms,^6,15,24^ affected the microbiome in this and other studies.^11,25^ Although we cannot disentangle causative factors without longitudinal sampling during travel,^26,27^ this evidence is suggestive of a temporary loss of colonisation resistance during travel.

We observed a significant enrichment of *E coli* strains in the gut after travel, with over two-thirds of travellers acquiring a new strain. Understanding risk factors for travel-associated *E coli* acquisition can help to disentangle the roles of local prevalence of drug resistance and individual exposure. For instance, the low rate of acquisition for the targeted AMR organisms in southern Africa suggests a lower prevalence of drug resistance, rather than a smaller exposure to bacterial reservoirs, based on the similar rates of *E coli* acquisition seen in this region compared with other regions. Our study highlights the potential utility of studying travellers as sentinels to identify AMR reservoirs and prevalence in countries that do not have a robust surveillance framework. We observed elevated levels of fluoroquinolone resistance-associated gene acquisition relative to an earlier study of Dutch travellers;^16,23^ this finding is also concordant with a recent study of travellers from China.^28^ Ongoing surveillance could robustly identify such temporal changes in resistance-acquisition patterns as global antibiotic consumption patterns and the prevalence of AMR organisms change.

We also found that *E coli* phylogroup A and B1 strains were commonly acquired during travel, whereas phylogroups B2 and D were dominant before travel. We observed little difference in phylogroup pattern across travel regions (appendix 1 p 10), consistent with faecal and wastewater sampling studies showing low correlation between geography and phylogroup structure.^29^ Travel-associated acquisition of phylogroup A might be explained by dietary exposures, since this phylogroup is commonly associated with food products.^30^ Further investigation is required to understand the implications of phylogroup A and B1 strain acquisition for traveller health.

Some results in this study were unexpected and require further investigation. First, pre-travel receipt of the injected typhoid vaccine was associated with a reduced risk of *E coli* acquisition. Cross-protection against the acquisition of enterotoxigenic *E coli* was previously shown for the oral cholera vaccine,^31^ but causality and a possible mechanism is unclear. We additionally found that reported consumption of unfiltered tap water was associated with a reduced risk of *E coli* acquisition in international travellers. Although this association was retained in multivariable models that adjusted for potential confounding factors, it might reflect a subset of travellers drinking water at locations with high sanitary standards, and engaging in low-risk behaviours.

Our study has limitations. Strain detection within metagenomic samples is limited by sequencing depth and the typically low relative abundance of *Enterobacteriaceae* species. Metagenomic strain detection at typical sequencing depths is probably associated with false negative results for low-abundance strains that might still be detected by culture-based methods. However, as this limitation applies equally to pre-travel and post-travel samples, we do not anticipate this to affect the estimated magnitude of strain acquisition. Furthermore, our previous culture-based analyses^15^ supported the finding of widespread *E coli* acquisition, as the majority of acquired targeted AMR organisms belonged to this species. We consolidated destinations by global regions but acknowledge that considerable heterogeneity in ESBL-producing organism prevalence, climate, sanitation, and diet exists within each region. Although our sample size was larger than previous metagenomic studies, statistical power remained insufficient to identify effects for specific regions or subgroups. Most travellers in this study were recruited from one site (Boston, MA, USA). Although outcomes among travellers recruited from smaller sites were broadly similar, our results might not be generalisable to the US traveller population. Finally, the collection of only pre-travel and post-travel samples prevents examination of the persistence of acquired AMR organisms and gut perturbation. We believe improved insights into risk factors for acquisition, rather than persistence, will be more valuable for designing interventions to limit the public health effects of the spread of AMR organisms, although future studies exploring persistence will be complementary to this goal.

Our study is the first detailed metagenomic evaluation of the gut dynamics of US international travellers, revealing frequent acquisition of novel *Enterobacteriaceae* strains and AMR-associated genes. Our ability to untangle resistant and general *E coli* acquisition here highlights a new model to evaluate the risk of, for example, ESBL-producing organism acquisition as a product of behavioural risk factors for *E coli* acquisition and local prevalence of ESBL-producing organisms. Our findings also highlight the potential utility of targeting preventive strategies to international travellers, including pre-travel education and minimising unnecessary antibiotic use during travel, with the aim of restricting the global dispersal of AMR.

## Supplementary Material

Appendix 1

Appendix 2

## Figures and Tables

**Table:** Baseline characteristics

	All participants
Age (years)	53 (10–21)
Travel duration (days)	15 (10–21)
Sex	
Female	161/267 (60%)
Male	106/267 (40%)
International travel in past year	122/194 (63%)
Antibiotics in past year	57/194 (29%)
Hospital admission in past year	12/193 (6%)
Enrolment site	
Boston, MA	222/267 (83%)
New York, NY	28/267 (11%)
Salt Lake City, UT	17/267 (6%)
Travel destination	
South America	49/267 (18%)
South Asia	42/267 (16%)
Southeast Asia	40/267 (15%)
East Africa	39/267 (15%)
Southern Africa	30/267 (11%)
West Africa	20/267 (8%)
Central America	19/267 (7%)
Other	28/267 (11%)
Targeted AMR organism colonisation	
Positive for any targeted AMR organism before travel	23/267 (9%)
Acquired ≥1 targeted AMR organism*	101/244 (41%)
Acquired ESBL-producing organisms*	99/244 (41%)
Acquired *mcr*-mediated colistin-resistant Enterobacterales*	18/244 (7%)
Acquired carbapenem-resistant Enterobacterales*	3/244 (1%)
Instances during travel	
Visiting friends or relatives	20/264 (8%)
Diarrhoea	88/262 (34%)
Taking antibiotics	30/260 (12%)

Data are median (IQR) or n/N (%). AMR=antimicrobial resistant. ESBL=extended-spectrum β-lactamase-producing organisms.

* The denominator is 244 so that percentages reflect those who were negative for target AMR organisms before travel.

## Data Availability

Metagenomic sequence data are available at the Sequence Read Archive under Bioproject PRJNA528511.

## Research in context

### Evidence before this study

We searched PubMed on April 4, 2023, for relevant previous publications using the search term: “international travel” AND (metagenome OR microbiome OR resistome). We identified six studies that used sequencing of faecal samples from international travellers, corresponding to four distinct traveller cohorts. Two of these six studies were based on 16S rRNA gene amplicon sequencing, allowing taxonomic profiling but not analysis of gene or strain content. Four studies used whole-metagenomic shotgun sequencing to profile traveller stool samples, one of which focused on longitudinal dynamics during travel in one destination country. Only one previous study examined the effect of travel on both the taxonomic profile of the gut microbiome and the resistome simultaneously. Previous studies were done on travellers based in Europe and southeast Asia, and three of the four cohorts examined included fewer than 100 participants.

### Added value of this study

This study used whole-metagenome sequencing to characterise the effect of travel on the gut microbiome and resistome simultaneously. We applied culture-independent metagenomic techniques to establish strain-level *Escherichia coli* dynamics, identifying high acquisition rates of *E coli* strains from specific phylogroups. Resistome analysis highlighted a significant increase in genes conferring resistance to a range of drug classes, including several genes previously classified as high risk from a public health perspective. Our findings build upon previous studies of extended-spectrum β-lactamase (ESBL) acquisition to highlight a wider range of travel-acquired antimicrobial-resistant organisms and *E coli* specifically. We found no evidence of colonisation resistance provided by the pre-travel microbiome.

### Implications of all the available evidence

We have shown that international travellers return with a wide range of antimicrobial-resistant organisms in the gut and elevated levels of *Enterobacteriaceae*, driven by the acquisition of novel *E coli* from phylogroups A and B1. This finding suggests that a narrow focus on ESBL acquisition is insufficient to quantify the public health risk posed by traveller acquisition of antimicrobial-resistant organisms. Via metagenomic analysis, we identified the broad acquisition of new *E coli* strains in the gut during travel, beyond just the ESBL *E coli* that are identified in culture-based approaches. Our results highlight that the acquisition of antimicrobial-resistant organisms by travellers is probably an interplay between the acquisition of novel bacterial strains in the gut and the prevalence of antibiotic resistance in these strains at the travel destination. Furthermore, our risk factor analysis suggests that efforts to reduce the rate of acquisition of antimicrobial-resistant organisms in travellers by altering behaviour (eg, promoting handwashing and avoiding unnecessary antibiotic use) might be more effective than interventions seeking to modulate the state of the pre-travel microbiome (eg, probiotics).
