# Supplementary material for: Gut microbiome perturbation, antibiotic resistance, and Escherichia coli strain dynamics associated with international travel: a metagenomic analysis
Source: Lancet Microbe. Author manuscript; Available in PMC 2023 Nov 27. (PMC10680401; doi:10.1016/S2666-5247(23)00147-7)
Supplement: Appendix 1 [file NIHMS1935677-supplement-Appendix_1.pdf]

THE LANCET
Microbe

Supplementary appendix 1

This appendix formed part of the original submission and has been peer reviewed. We post it as supplied by the authors.

Supplement to: Worby CJ, Sridhar S, Turbett SE, et al. Gut microbiome perturbation, antibiotic resistance, and *Escherichia coli* strain dynamics associated with international travel: a metagenomic analysis. *Lancet Microbe* 2023; published online Sept 13. https://doi.org/10.1016/S2666-5247(23)00147-7.

Appendix to “Gut microbiome perturbation, antibiotic resistance, and E. coli strain dynamics associated with international travel: a metagenomic analysis”

Colin J. Worby, Ph.D., Sushmita Sridhar, Ph.D., Sarah E. Turbett, M.D., Margaret V. Becker, B.S., Lucyna Kogut, B.S., Vanessa Sanchez, B.S., Ryan A. Bronson, M.S., Sowmya R. Rao, Ph.D., Elizabeth Oliver, R.N., Allison Taylor Walker, Ph.D., Maroya Spalding Walters, Ph.D., Paul Kelly, M.D., Daniel T. Leung, M.D., Mark C. Knouse, M.D., Stefan H.F. Hagmann, M.D., Prof. Jason B. Harris M.D., Edward T. Ryan M.D., Ashlee M. Earl, Ph.D., Regina C. LaRocque M.D., M.P.H.

Table of Contents

Supplementary Methods 2
References 3
Traveler Questionnaire 4
Supplemental Figures 8
Supplementary Table Legends 12

Supplementary Methods

DNA extraction, library construction and sequencing. We performed extractions on stool samples using the PowerFecal DNA/RNA kit (QIAGEN; Hilden, Germany). Metagenomic libraries were prepared using the Nextera XT DNA Library Preparation kit (Illumina; San Diego, CA). Sequencing was performed on an Illumina NovaSeq 6000 with 151 bp paired-end reads to yield a median of 14 million paired-end reads per sample. Data were analyzed using the Broad Picard Pipeline (https://broadinstitute.github.io/picard).

Metagenomics. Reads were processed with KneadData (1) to remove adapter sequence and trim low base qualities, as well as to remove human-derived sequences. A median of 14 million reads per sample were available after filtering. Bacterial community composition was determined using Kraken v2.0.7 and Bracken (2). Shannon diversity and Bray Curtis distances were calculated using species-level profiles (3).

E. coli strain analysis. We used StrainGE to characterize the *E. coli* population within each sample at strain level (4). 929 complete RefSeq *Escherichia* chromosomes were downloaded in 2019 and clustered at a Jaccard k-mer similarity of 0.9, corresponding to 99.8% average nucleotide identity, resulting in 361 reference genomes. Strains were considered 'acquired' if they were present in post-travel samples, but not in pre-travel samples.

Resistome analysis. Resistance analysis on the metagenomic samples was conducted using ShortBRED(5). A ShortBRED marker database was built from the CARD resistance database protein homolog model (6) using a 95% cluster identity cutoff. Uniref90 (7) was used as the reference masking protein database. Metagenomic reads were mapped to the ShortBRED marker sequence database to determine relative abundance of AMR genes (5). ARGs were defined as 'high-risk' if they belonged to the 'Rank I - current threat' category in the classification from Zhang et al. (8). High-risk ARGs were deemed present in a metagenomic sample if the representative marker(s) for their cluster were identified.

Statistical models. We fit logistic and linear regression models using the *lme4* (v1.1-30) package (9) and the *lm* function in R (v4.0.3). For each outcome and variable, we first fit unadjusted regression models (adjusting for sequencing depth when considering microbiome-related outcomes and pre-travel diversity when considering perturbation). For multivariable models, variables were selected based on significance in unadjusted models, together with previous literature and biological plausibility. Selected variables and their estimated coefficients for multivariable models are in Supplementary Tables 2-5. We report odds ratios and 95% CIs for all outcomes, with the exception of microbiome perturbation, where we estimate the change in Bray Curtis dissimilarity.

Multiple imputation was applied for multivariable models including variables with >5% missing data. Two variables had missing values for 33% of cases (travel in previous 12 months, antibiotic use in previous 12 months). For multivariable models in which these variables were included, multiple imputation was implemented using the R package mice (v 3.15.0) with default settings. Taxa relative abundances were log-transformed, with zero values assigned the

smallest detected relative abundance across all other taxa. Only taxa detected in at least 10% of all samples were included.

We aggregated destinations into geographic regions as defined by the United Nations Statistics Division for the purpose of statistical analyses. We defined visiting friends or relatives (VFR) travel as traveling to a low or middle income (as defined by United Nations Human Development Index) region of origin of the traveler or the traveler's parents to visit friends and relatives.

References

1. McIver LJ, Abu-Ali G, Franzosa EA, Schwager R, Morgan XC, Waldron L, et al. bioBakery: a meta'omic analysis environment. Bioinformatics. 2018;34(7):1235-7.
2. Wood DE, Lu J, Langmead B. Improved metagenomic analysis with Kraken 2. Genome Biol. 2019;20(1):257.
3. Paradis E, Claude J, Strimmer K. APE: Analyses of Phylogenetics and Evolution in R language. Bioinformatics. 2004;20(2):289-90.
4. van Dijk LR, Walker BJ, Straub TJ, Worby CJ, Grote A, Schreiber HLt, et al. StrainGE: a toolkit to track and characterize low-abundance strains in complex microbial communities. Genome Biol. 2022;23(1):74.
5. Kaminski J, Gibson MK, Franzosa EA, Segata N, Dantas G, Huttenhower C. High-Specificity Targeted Functional Profiling in Microbial Communities with ShortBRED. PLoS Comput Biol. 2015;11(12):e1004557.
6. Alcock BP, Raphenya AR, Lau TTY, Tsang KK, Bouchard M, Edalatmand A, et al. CARD 2020: antibiotic resistome surveillance with the comprehensive antibiotic resistance database. Nucleic Acids Res. 2020;48(D1):D517-D25.
7. Suzek BE, Huang H, McGarvey P, Mazumder R, Wu CH. UniRef: comprehensive and non-redundant UniProt reference clusters. Bioinformatics. 2007;23(10):1282-8.
8. Zhang AN, Gaston JM, Dai CL, Zhao S, Poyet M, Groussin M, et al. An omics-based framework for assessing the health risk of antimicrobial resistance genes. Nat Commun. 2021;12(1):4765.
9. Bates D, Mächler M, Bolker B, Walker S. Fitting Linear Mixed-Effects Models Using lme4. Journal of Statistical Software. 2015;67(1):1-48.
10. D'Souza AW, Boolchandani M, Patel S, Galazzo G, van Hattem JM, Arcilla MS, et al. Destination shapes antibiotic resistance gene acquisitions, abundance increases, and diversity changes in Dutch travelers. Genome Med. 2021;13(1):79.

Traveler Questionnaire

Surveillance System for the Introduction of Antimicrobial Resistant (AMR) Organisms into the U.S. Following International Travel

Travel Activities

1. When did you depart on your trip? ___________________________ (date)

2. When did you return from your trip? ___________________________ (date)

3. How many countries did you visit?

4. What was the duration of your stay in each country?

   Country ___________________________ # of days ________________

   Country ___________________________ # of days ________________

   Country ___________________________ # of days ________________

   Country ___________________________ # of days ________________

   Country ___________________________ # of days ________________

   Country ___________________________ # of days ________________

   Country ___________________________ # of days ________________

5. Did you participate in any of the following activities during your trip overseas? (If the answer is yes, please indicate in which country.)

   drinking unpurified tap water Yes/No/Don't Know in which country?__________

   eating food from a street vendor Yes/No/Don't Know in which country? ___________

   eating in the home of a family or friend Yes/No/Don't Know in which country? ___________

   eating uncooked vegetables Yes/No/Don't Know in which country? ___________

   eating raw or undercooked red meat Yes/No/Don't Know in which country? ___________

   eating raw or undercooked fish Yes/No/Don't Know in which country? ___________

   eating unpeeled fruit or

   drinking raw fruit juice or smoothies Yes/No/Don't Know in which country? ___________

swimming or going into a lake, river,

stream or natural spring Yes/No/Don't Know in which country? __________

swimming or going into a pool,

waterpark, hot tub/spa, or fountain Yes/No/Don't Know in which country? __________

swimming or going into the ocean Yes/No/Don't Know in which country? __________

working Yes/No/Don't Know in which country? __________

visiting a health care facility Yes/No/Don't Know in which country? __________

visiting a farm, petting zoo or household where animals were present

Yes/No/Don't Know in which country? __________

(which animals? ____________________)

Travel Related Diarrhea

We define diarrhea as 3 or more episodes of loose stool in a 24 hour period.

5. Did you experience any diarrhea on your trip or within 3 days of your return home? Yes/No

6. If yes, when did the diarrhea begin?
   During the trip? Yes/No
   During the return trip home? Yes/No
   After returning home? Yes/No

7. If yes, for how many days did you experience diarrhea?
   ______________________days

8. If yes, were these days of diarrhea continuous or episodic? Continuous/episodic

9. If yes, in which country did your symptoms start? ______________________

10. Did you find the diarrhea distressing? Yes/No

11. Did you find the diarrhea incapacitating? Yes/No

12. Did you have any blood in your stool along with diarrhea? Yes/No

13. Did you have fever along with the diarrhea? Yes/No

14. Did you have to miss any planned activities because of diarrhea? Yes/No

15. What was the greatest number of episodes of diarrhea that you experienced in a single day?

    3-5

    6 or more

16. If you never had 3 or more episodes of loose stool in a 24 hour period, did you have a milder case of diarrhea on your trip? Yes/No

Treatment for Diarrhea

17. What medication/treatment did you take for your diarrhea?

    Ciprofloxacin Yes/No

    Azithromycin Yes/No

    Rifaximin Yes/No

    Other antibiotic (name: ______________) Yes/No

    Imodium Yes/No

    Lomotil Yes/No

    Pepto-Bismol Yes/No

    Oral rehydration salts/Pedialyte Yes/No

    Natural product (name:______________) Yes/No

    Other medicine (name: ____________) Yes/No

    The traveler took no medication or treatment

18. Where did you obtain this medication/treatment?
    Brought from the US Yes/No
    Purchased overseas Yes/No
    In which country? _______________________

19. Did you seek medical attention for diarrhea? Yes/No

    If yes, where did you seek medical attention? (Check all that apply)

Pharmacy

Hotel doctor

Outpatient setting (clinic, urgent care center, emergency department, hospital clinic)

describe: ______________________

Inpatient setting (hospital, urgent care, short stay observation, ER observation)

describe: _______________________

Other (describe: ___________)

20. Have your bowel movements returned to your normal? Yes/No

21. Were any of your travel companions ill with diarrhea on your trip? Yes/No

Treatment for Other Travel-Related Health Issues

22. Did you seek health care for any other reason during your trip? Yes/No

What reason? ____

What setting? ____

What treatment? ____

23. Did you take antibiotics for any other reason during your trip? Yes/No

Which antibiotic(s)? ____

Where did you obtain this medication/treatment?

Brought from the US Yes/No

Purchased overseas Yes/No

In which country? _______________________

24. Did you take malaria chemoprophylaxis during your trip? Yes/No

Which medication? ____

Supplemental Figures

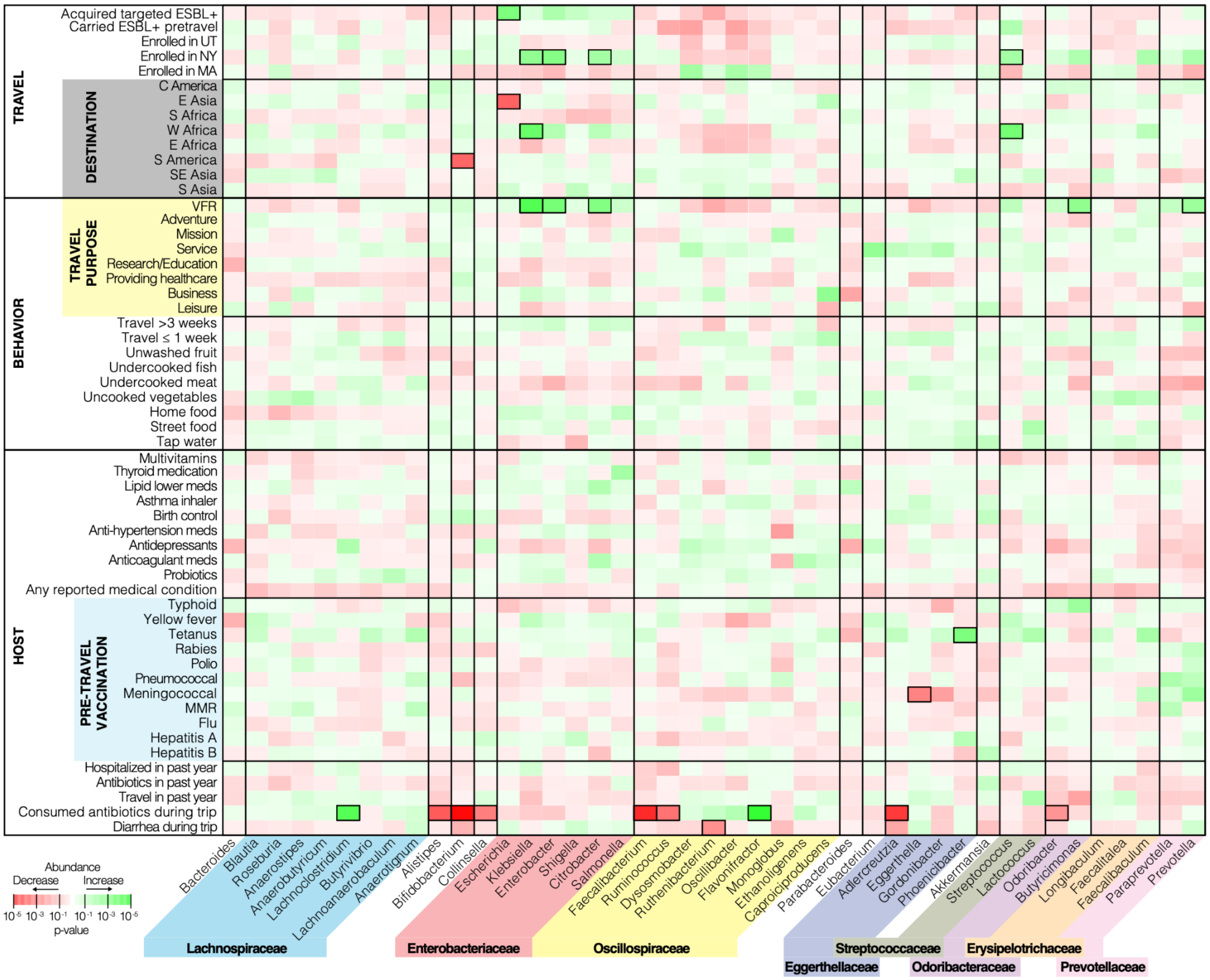

Supplementary Figure 1. Associations between genus relative abundances and travel, host and behavioral variables. For all genera within the fifteen most common bacterial families, we fit models for change in relative abundance as a function of each of the travel, host, and behavioral variables on the left. A colored box denotes each variable-genus pair, colored green if the variable is associated with an increase in relative abundance, red if associated with a decrease. Bolder colors denote lower p values; all pairs with p values < 0.05 after multiple testing correction are highlighted with a black border.

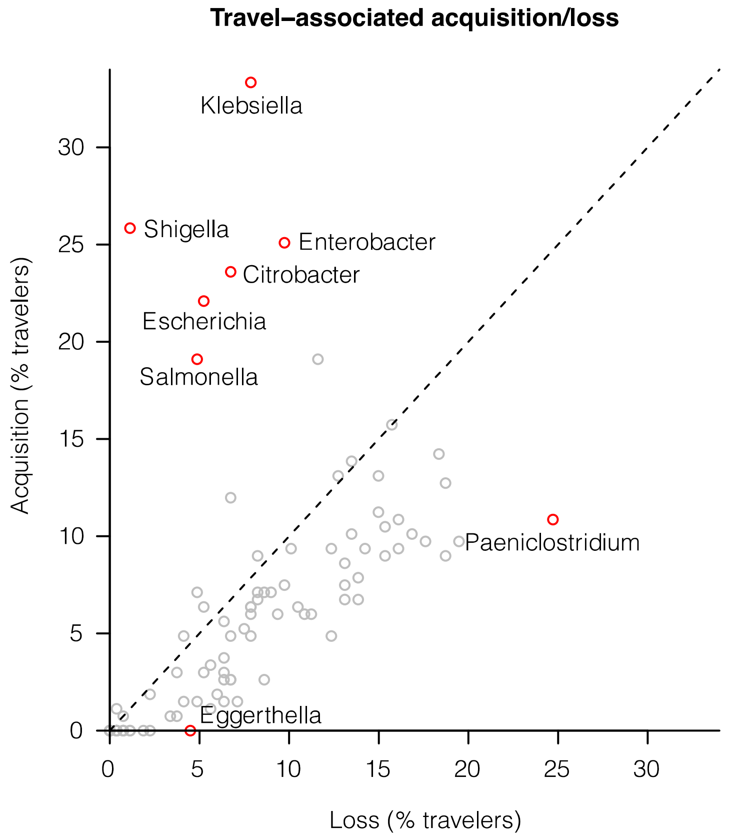

Supplementary Figure 2. Acquisition and loss of bacterial genera associated with international travel. For each genus present in at least 10% of samples, the proportion of travellers with an observed acquisition (genus detected only post-travel) or loss (genus detected only pre-travel). Red labelled points denote genera with significant skew.

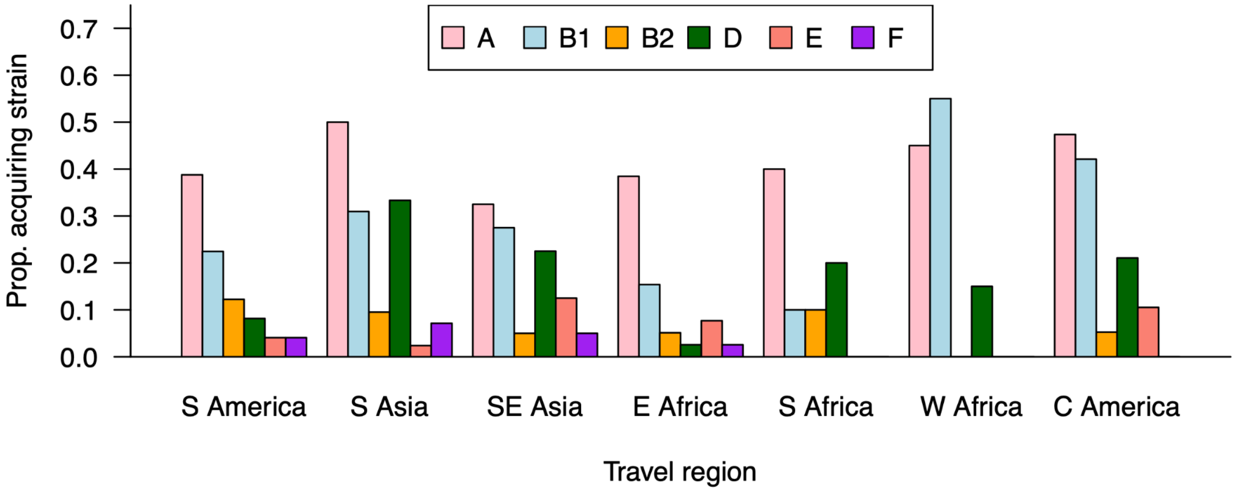

Supplementary Figure 3. Acquired *E. coli* strain phylogroups. For each of the most common travel regions, bars denote the proportion of travelers acquiring *E. coli* strains belonging to particular phylogroups, encoded by colors.

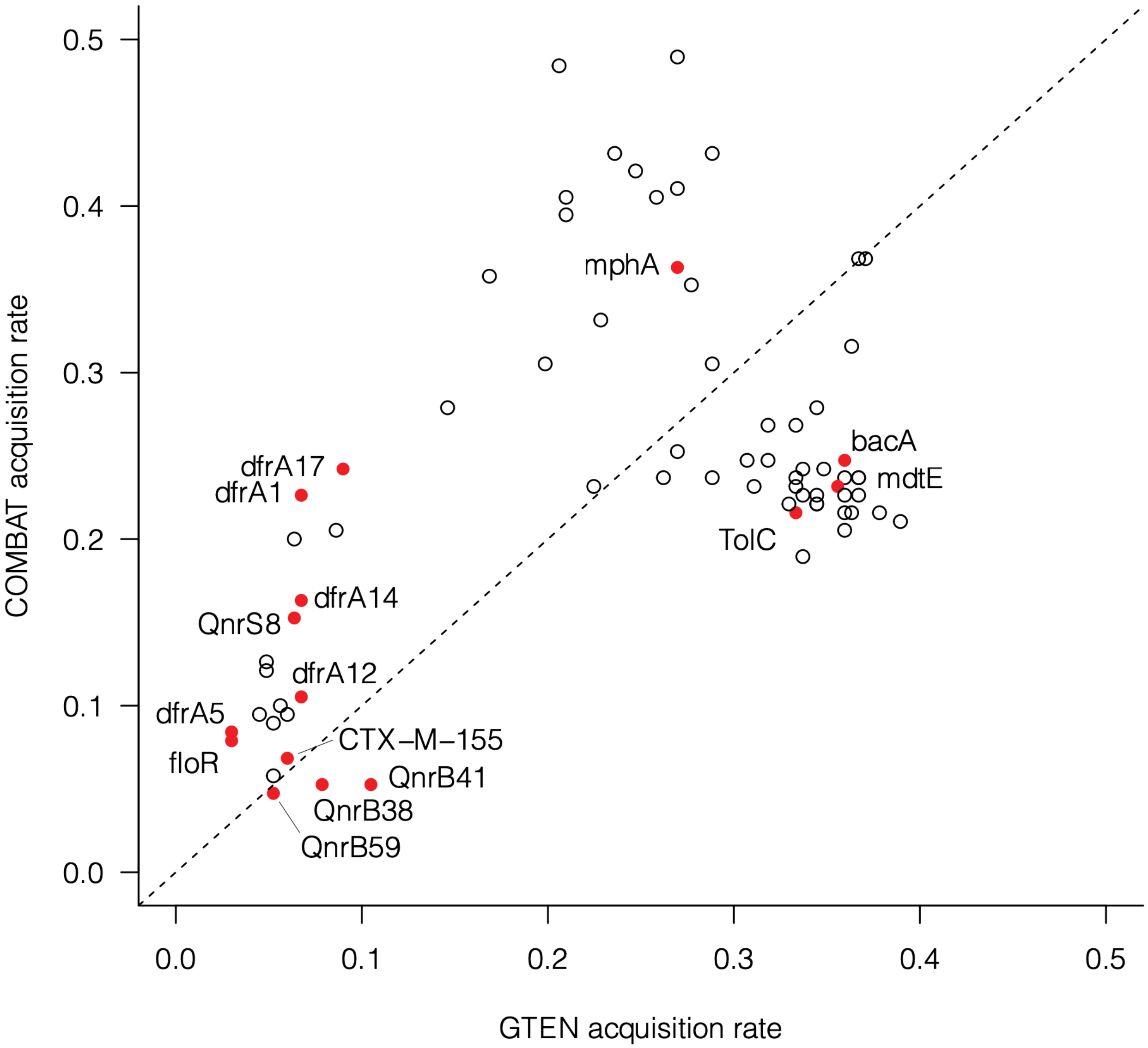

Supplementary Figure 4. COMBAT vs. GTEN ARG acquisitions. Acquisition rates of ARGs found to be acquired at a significant rate (Binomial test vs. equal loss) in our study (2018-2020) and/or the COMBAT study (2013-2014) of Dutch travelers (10) are compared. High risk acquisitions are marked as filled points and labeled.

Supplementary Table Legends

Supplementary Table 1. Summary of traveler variables. For binary variables, the number (n) and percentage of travelers with a positive response are given; median and interquartile range (IQR) is provided for continuous variables.

Supplementary Table 2. Regression model results for targeted AMR organism acquisition. For all binary variables considered, the number and proportion overall, and by targeted AMR organism status, are provided. For all variables, the estimated log odds ratio, confidence intervals (CIs) and p values are reported for the individual unadjusted models as well as the multivariable model.

Supplementary Table 3. Regression model results for diarrhea. For all binary variables considered, the number and proportion overall, and by diarrhea status, are provided. For all variables, the estimated log odds ratio, confidence intervals (CIs) and p values are reported for the individual unadjusted models as well as the multivariable model.

Supplementary Table 4. Regression model results for gut microbiome perturbation. For all binary variables considered, the number and proportion of positive responses overall are provided. For all variables, the estimated coefficients, confidence intervals (CIs) and p values are reported for the individual models (including sequencing depth and initial gut diversity as covariates) as well as the multivariable model.

Supplementary Table 5. Regression model results for *E. coli* strain acquisition. For all binary variables considered, the number and proportion overall, and by *E. coli* acquisition status, are provided. For all variables, the estimated log odds ratio, confidence intervals (CIs) and p values are reported for the individual models (including sequencing depth as a covariate) as well as the multivariable model.

Supplementary Table 6. Pre- and post-travel ARG burden by travel region. For each of the six most common travel destinations, the pre- and post-travel ARG burden, measured by reads per kilobase per million reads (RPKM) is provided.

Supplementary Table 7. Traveler ARG dynamics. For all detected ARGs, details from the CARD database, classification as a ‘high risk’ ARG, correlation with the relative abundance of *E. coli*, as well as the proportion of travelers who lost or acquired the ARG are provided, along with the p values for bias towards gain or loss (exact Binomial test).
